# Supplementary material for: The Underlying Physicochemical Properties and Starch Structures of indica Rice Grains with Translucent Endosperms under Low-Moisture Conditions
Source: Foods. 2022 May 10;11(10):1378. doi: 10.3390/foods11101378 (PMC9141583; doi:10.3390/foods11101378)
Supplement: Supplementary file 1 [file foods-11-01378-s001.zip › foods-1698013-supplementary.pdf]

foods

MDPI

Article

The Underlying Physicochemical Properties and Starch Structures of *indica* Rice Grains with Translucent Endosperms under Low-Moisture Conditions

Fei Chen[1], Yan Lu [1], Lixu Pan [1], Xiaolei Fan [1], Qianfeng Li [1], Lichun Huang [1], Dongsheng Zhao [1], Changquan Zhang [1,2] and Qiaoquan Liu [1,2,*]

Table S1. Relative translucence level of rice grain transparency at gradient drying time

Rice varieties	2h	4h	6h	8h	12h	24h
YFN	5.433±0.222a	6.062±0.335a	6.143±0.43a	7.619±0.191a	6.350±0.716a	7.613±0.102a
28Z	4.89±0.143b	5.131±0.383bc	5.687±0.162a	6.09±0.641b	5.931±0.452ab	6.421±0.128b
HHZ	4.21±0.169ab	4.471±0.271c	4.654±0.103b	4.846±0.125c	5.228±0.307b	5.749±0.141c
9311	5.437±0.18a	5.507±0.403ab	4.632±0.573b	5.602±0.139b	5.826±0.321ab	6.220±0.200b

Values are means ± SD, (n = 3). Values in the same column with different forms of letters indicate significant differences determined by Student's *t*-test (p <0.05).

Table S2. The RVA spectrum characteristic values of different rice samples extracted from RVA curves

Rice varieties	Peak 1 (cP)	Trough 1 (cP)	Breakdown (cP)	Final Viscosity (cP)	Setback (cP)
YFN	2530b	1335c	1195b	1643b	-887d
28Z	3261a	1860b	1401a	3046a	-215c
HHZ	3279a	2034a	1245b	3482a	203b
9311	3037a	2243a	794c	3448a	411a

Values are means ± SD, (n = 3). Values in the same column with different forms of letters indicate significant differences determined by Student's *t*-test (p <0.05).

Table S3. The thermodynamic properties of rice starch from different samples determined by DSC

Rice varieties	ΔH (J/g)	T_o (°C)	T_p (°C)	T_c (°C)
YFN	16.76±0.1a	72.2±0.35a	77.53±0.29a	86.27±0.29a
28Z	11.84±0.81b	64.03±0.25b	68.57±0.5b	78.93±0.23b
HHZ	12.37±0.17b	63.5±0.17c	68.63±0.15b	75.83±0.40c
9311	11.64±0.66b	62.7±0.17d	67.37±0.06c	74.33±0.06d

Values are means ± standard deviations (n = 3). Values in the same column with different forms of letters indicate significant differences determined by Student's *t*-test (p <0.05).

Table S4. ATR-FTIR and SAXS parameters of starch

Rice varieties	1047/1022 (cm^{-1})	1022/995 (cm^{-1})	I_{max} (counts)	ΔS ($Å^{-1}$)	S_{max} ($Å^{-1}$)	D (nm)
YFN	0.713±0.012a	1.541±0.014b	333.143±5.745a	0.018±0.002b	0.044±0.003b	13.545±0.200a
28Z	0.663±0.029ab	1.361±0.020c	157.963±6.429bc	0.018±0.003ab	0.064±0.006a	9.168±0.208b
HHZ	0.662±0.015ab	1.152±0.015d	160.850±5.000b	0.020±0.002ab	0.063±0.003a	9.372±0.104b
9311	0.615±0.050b	1.707±0.035a	146.939±6.429c	0.022±0.003a	0.062±0.005a	9.384±0.076b

Values are means ± standard deviations (n = 3). Values in the same column with different forms of letters indicate significant differences determined by Student's *t*-test (p <0.05). I_{max}: Peak intensity; S_{max}: Peak position; ΔS: Peak width at half maximum; D: Bragg spacing ($2\pi/S_{max}$).

Table S5. Starch fine structure parameters were extracted from relative molecular weight distribution curves and chain length distribution curves

Rice varieties	AM (%)	LAP (%)	SAP (%)	ΣDP (6-12) (%)	ΣDP (13-24) (%)	ΣDP (25-37) (%)	ΣDP (>34) (%)
YFN	4.64±0.74d	24.3±0.02a	75.9±0.003a	16.50±0.05d	36.78±0.11b	18.16±0.081a	27.62±0.11a
28Z	13.05±0.14c	22.3±0.01b	62.3±0.006c	20.45±0.05b	39.73±0.21a	17.59±0.11c	21.00±0.05b
HHZ	16.16±0.22a	21.7±0.01b	61.6±0.007c	20.09±0.05c	39.81±0.11a	17.80±0.09b	21.02±0.11b
93-11	15.11±0.18b	20.8±0.00c	64.4±0.01b	20.64±0.09a	39.98±0.07a	17.65±0.05bc	20.59±0.03c

Values are means ± SD, (n = 3). Values in the same column with different forms of letters indicate significant differences determined by Student's *t*-test (p <0.05). AM, Amylose content; LAP, Long-chain amylopectin; SAP, Short-chain amylopectin； DP, Degree of polymerization.
